# Supplementary material for: Nest-site selection, reproductive ecology and shifts within core-use areas of Black-necked Cranes at the northern limit of the Tibetan Plateau
Source: PeerJ. 2017 Jan 31;5:e2939. doi: 10.7717/peerj.2939 (PMC5289107; doi:10.7717/peerj.2939)
Supplement: Table S1 [file peerj-05-2939-s001.docx]

| **yy/mm/dd** | **number** | **Adult*** | **sub-adult**** | **Chicks***** |
| --- | --- | --- | --- | --- |
| **2013/4/5** | **12** | **12** |  |  |
| **2013/4/20** | **40** | **30** | **10** |  |
| **2013/5/10** | **72** | **72** | **0** | **0** |
| **2013/5/30** | **77** | **74** | **3** | **0** |
| **2013/6/10** | **98** | **76** | **9** | **13** |
| **2013/7/15** | **119** | **80** | **15** | **24** |
| **2013/8/20** | **123** | **84** | **14** | **25** |
| **2013/9/15** | **117** | **84** | **13** | **20** |
| **2013/10/15** | **149** | **86** | **35** | **28** |
| **2013/11/5** | **0** | **0** | **0** | **0** |
| **2014/3/28** | **5** | **4** | **1** | **0** |
| **2014/3/30** | **6** | **6** | **0** | **0** |
| **2014/4/2** | **10** | **10** | **0** | **0** |
| **2014/4/7** | **16** | **16** | **0** | **0** |
| **2014/4/10** | **37** | **32** | **5** | **0** |
| **2014/4/13** | **39** | **38** | **1** | **0** |
| **2014/4/16** | **75** | **62** | **13** | **0** |
| **2014/4/20** | **78** | **74** | **4** | **0** |
| **2014/4/25** | **76** | **76** | **0** | **0** |
| **2014/4/29** | **77** | **76** | **1** | **0** |
| **2014/5/4** | **72** | **72** | **0** | **0** |
| **2014/5/10** | **71** | **62** | **9** | **0** |
| **2014/5/25** | **77** | **74** | **3** | **0** |
| **2014/6/8** | **92** | **76** | **8** | **8** |
| **2014/7/18** | **143** | **92** | **21** | **30** |
| **2014/8/2** | **131** | **82** | **29** | **20** |
| **2014/8/20** | **123** | **84** | **14** | **25** |
| **2014/9/5** | **114** | **72** | **17** | **25** |
| **2014/9/14** | **127** | **74** | **28** | **25** |
| **2014/9/23** | **134** | **76** | **30** | **28** |
| **2014/10/15** | **148** | **80** | **42** | **26** |
| **2014/10/21** | **5** | **3** | **1** | **1** |
| **2014/10/29** | **0** | **0** | **0** | **0** |
| **2015-3-30** | **6** | **6** | **0** | **0** |
| **2015/4/5** | **20** | **12** | **8** | **0** |
| **2015/4/10** | **17** | **16** | **1** | **0** |
| **2015/4/15** | **27** | **26** | **1** | **0** |
| **2015/4/20** | **76** | **68** | **8** | **0** |
| **2015/4/25** | **89** | **76** | **13** | **0** |
| **2015/4/30** | **86** | **70** | **16** | **0** |
| **2015/5/5** | **90** | **76** | **14** | **0** |
| **2015/5/17** | **110** | **78** | **32** | **0** |
| **2015/5/30** | **100** | **82** | **14** | **4** |
| **2015/6/10** | **113** | **82** | **17** | **14** |
| **2015/6/20** | **134** | **80** | **12** | **42** |
| **2015/6/30** | **135** | **80** | **15** | **40** |
| **2015/7/15** | **138** | **82** | **24** | **32** |
| **2015/7/30** | **126** | **80** | **16** | **30** |
| **2015/8/15** | **130** | **84** | **19** | **27** |
| **2015/8/31** | **119** | **82** | **12** | **25** |
| **2015/9/15** | **129** | **84** | **25** | **20** |
| **2015/9/30** | **130** | **80** | **30** | **20** |
| **2015/10/10** | **129** | **84** | **25** | **20** |
| **2015/10/25** | **26** | **20** | **0** | **6** |
| **2015/11/5** | **8** | **6** | **0** | **2** |
| **2015/11/15** | **0** | **0** | **0** | **0** |

*The ‘‘adult’’ category included both mature breeding cranes and nonbreeding cranes that were always observed as a pair. **The ‘‘subadult’’ category included returning nonbreeding juveniles, which were always present in a flock. ***The ‘‘chick’’ category included chicks hatched in 2013, 2014 and 2015, which were easily distinguishable from adults and subadults in the hatching year by their sizes, heads and neck plumage.
