# Supplementary figures and images for: Nest-site selection, reproductive ecology and shifts within core-use areas of Black-necked Cranes at the northern limit of the Tibetan Plateau
Source: PeerJ. 2017 Jan 31;5:e2939. doi: 10.7717/peerj.2939 (PMC5289107; doi:10.7717/peerj.2939)

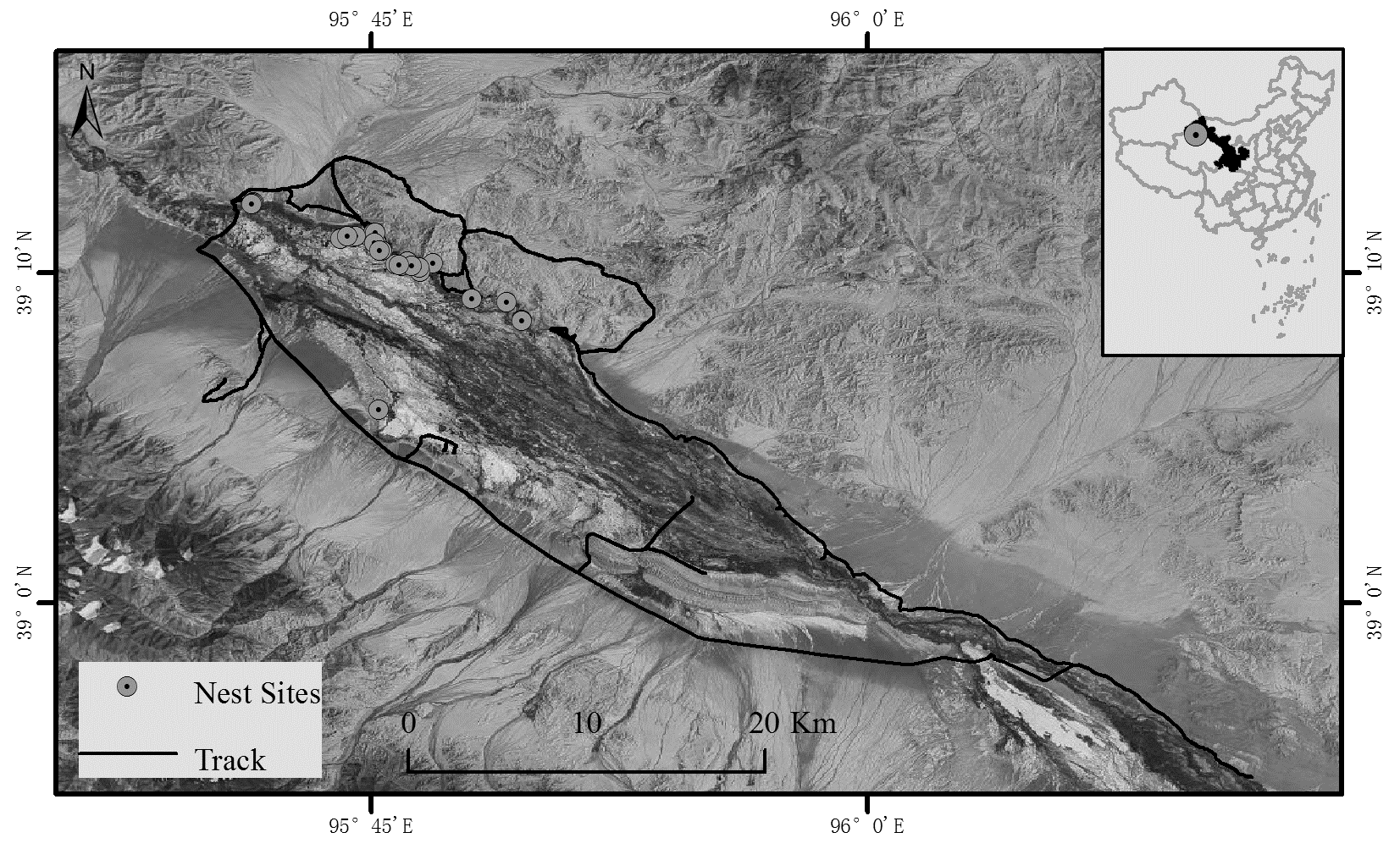

Supplement: Figure S1 — Black line: Black-necked Crane survey route. Grey circles with dots: locations of 29 monitored Black-necked Crane nests on the main Yanchiwan National Nature Reserve. [file peerj-05-2939-s002.png]

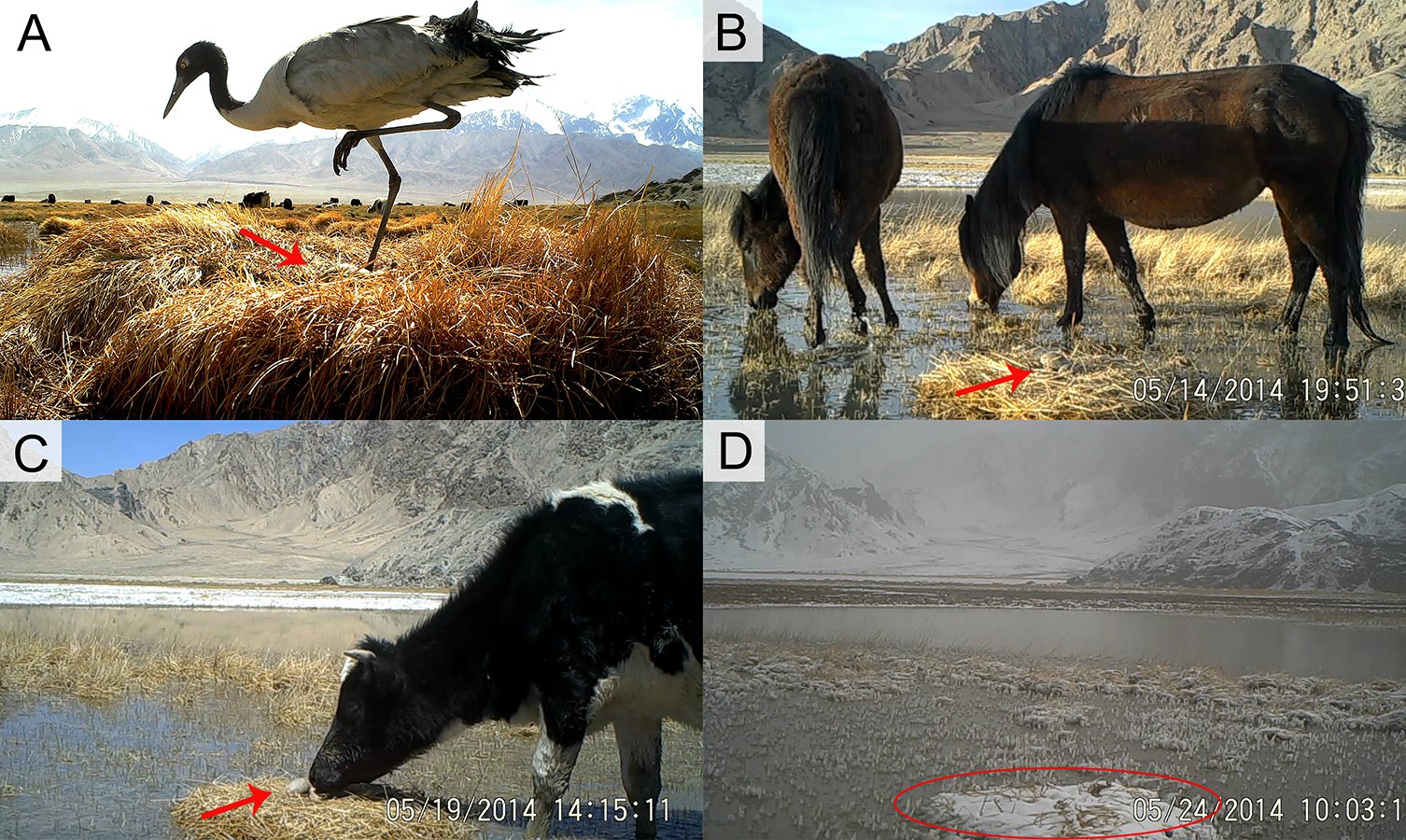

Supplement: Figure S2 — (A) Black-necked Crane on the nest with a herd of livestocks, (B) two horses near the nest, (C) A cow is sniffing eggs and (D) nest abandoned due to disturbance from livestocks. (A) Photo taken by LX Zhang and (B), (C) and (D) still photos captured from video footage at nests of Black-necked Cranes. [file peerj-05-2939-s003.png]

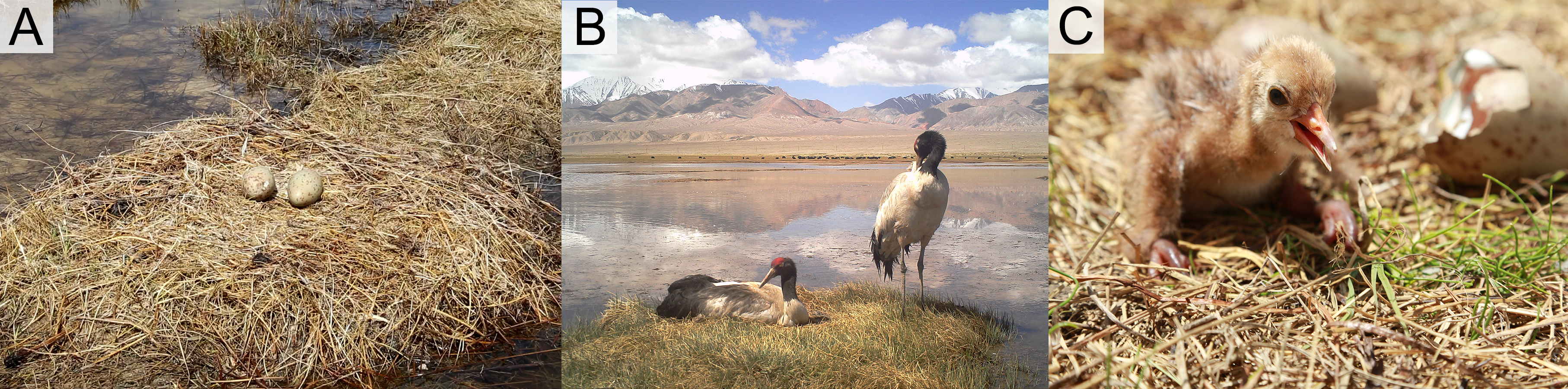

Supplement: Figure S3 — (A) two eggs on the haystack nest during parents’ incubation break (photo taken by LX Zhang), (B) one parent on the nest accompanied by the other parent (still photo captured from video footage at nests of Black-necked Cranes) and (C) a recently hatched chick (photo taken by LX Zhang). [file peerj-05-2939-s004.png]

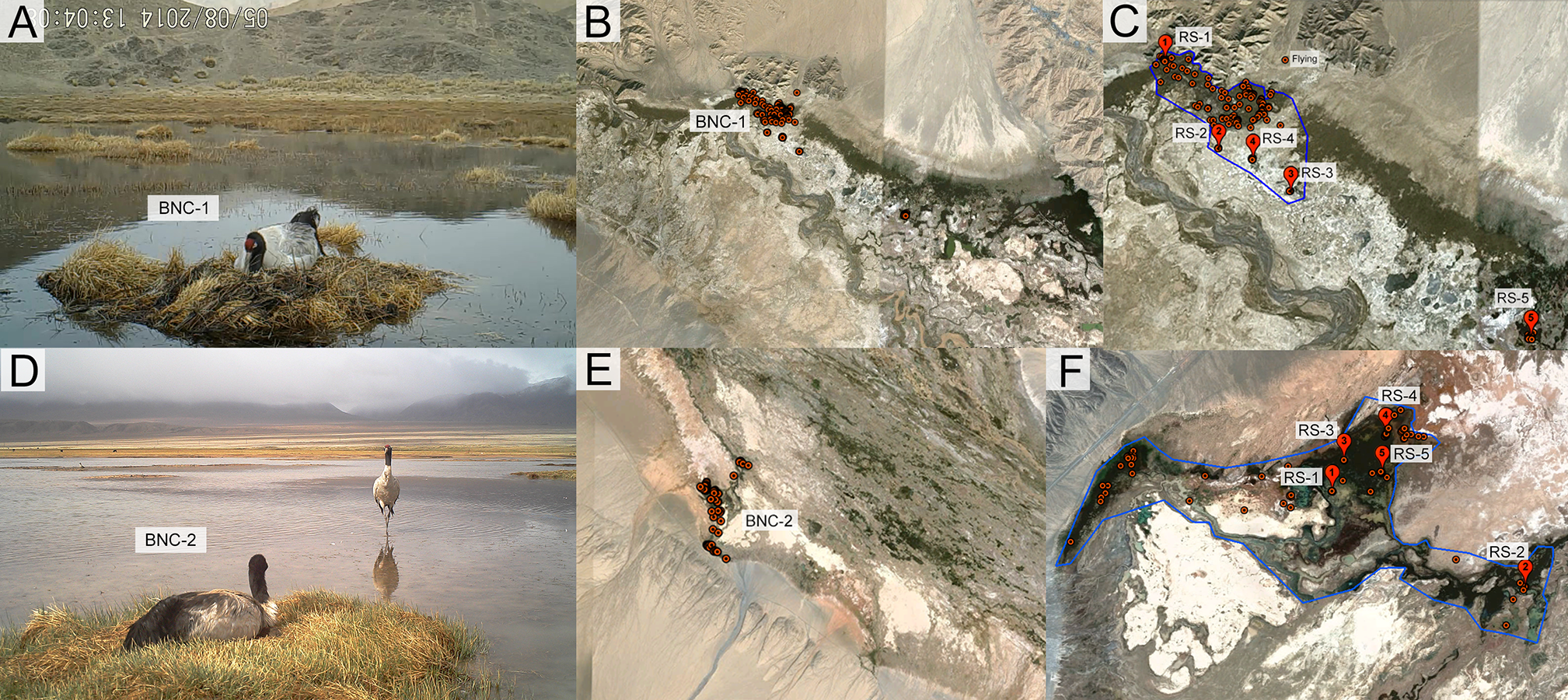

Supplement: Figure S4 — (A) View of BNC-1 nest environment, (B) active spots (red points) from BNC-1, (C) BNC-1 active range and different roosting shifts before migration, (D) view of BNC-2 nest environment, (E) active spots (red points) from BNC-2 and (F) BNC-2 active range roosting shifts before migration. [file peerj-05-2939-s005.png]

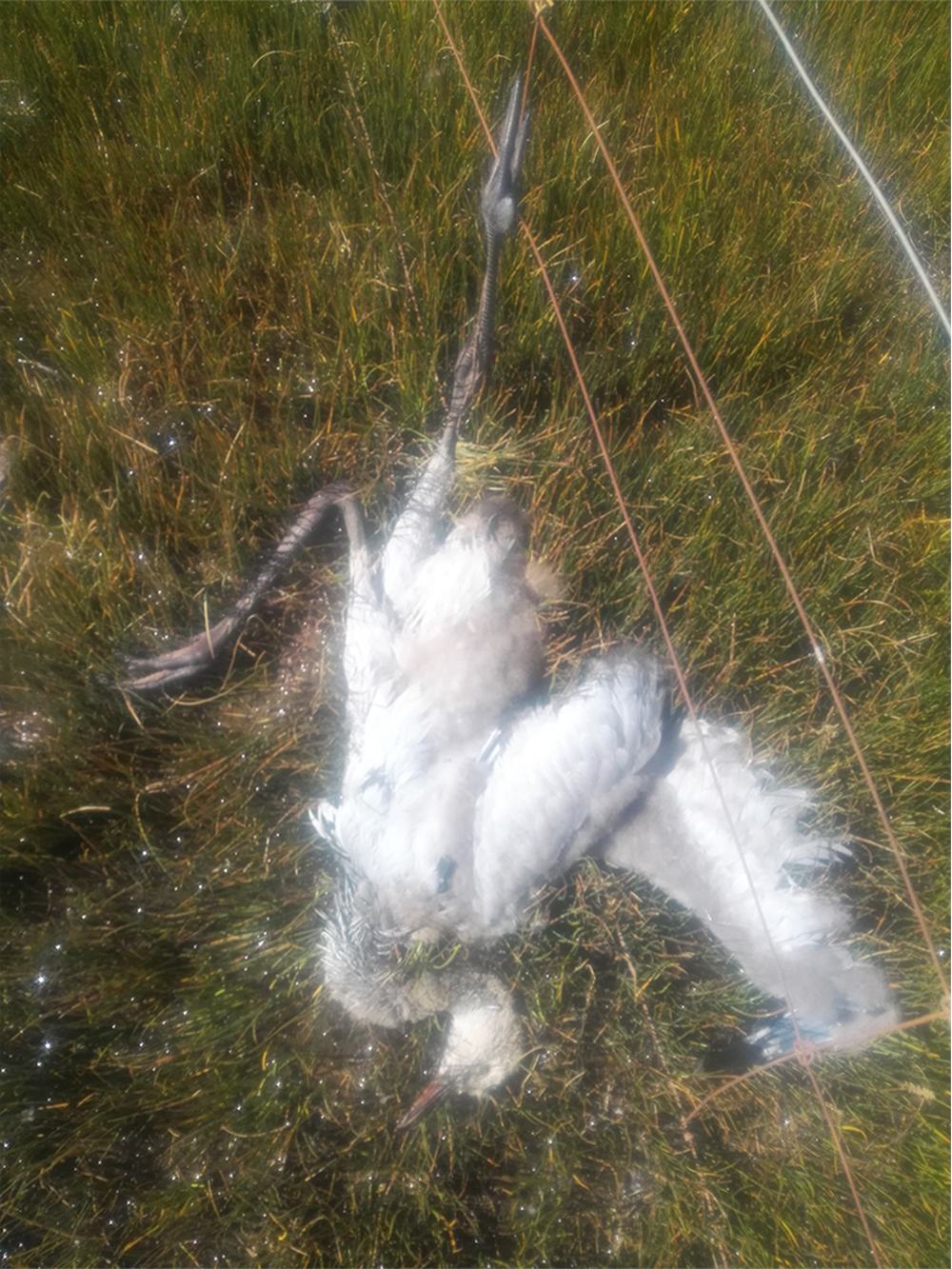

Supplement: Figure S5 — Photo taken by LX Zhang. [file peerj-05-2939-s006.png]

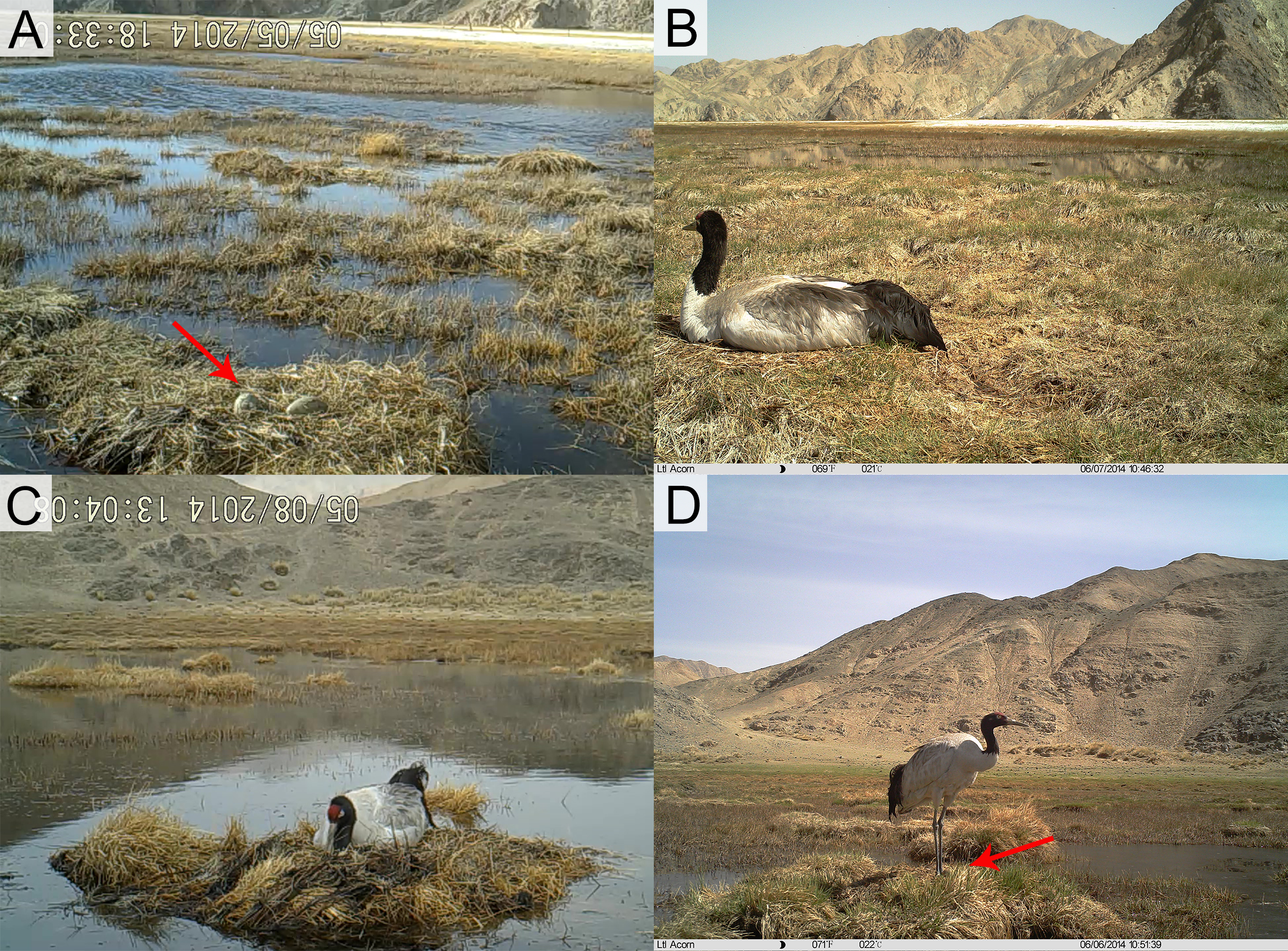

Supplement: Figure S6 — (A) & (B) Black-necked crane nest in a marsh where (A) = May 5 and (B) = June 6, (C) & (D) Black-necked crane nest in a pond where (C) = May 8 and (D) June 7 (all from still photos captured from video footage at nests of Black-necked Crane). [file peerj-05-2939-s007.png]
